# Lay Definitions of Happiness across Nations: The Primacy of Inner Harmony and Relational Connectedness

**DOI:** 10.3389/fpsyg.2016.00030

**Published:** 2016-01-26

**Authors:** Antonella Delle Fave, Ingrid Brdar, Marié P. Wissing, Ulisses Araujo, Alejandro Castro Solano, Teresa Freire, María Del Rocío Hernández-Pozo, Paul Jose, Tamás Martos, Hilde E. Nafstad, Jeanne Nakamura, Kamlesh Singh, Lawrence Soosai-Nathan

**Affiliations:** ^1^Department of Pathophysiology and Transplantation, University of MilanoMilan, Italy; ^2^Department of Psychology, University of RijekaRijeka, Croatia; ^3^Africa Unit for Transdisciplinary Health Research, North-West UniversityPotchefstroom, South Africa; ^4^School of Arts, Sciences and Humanities, University of São PauloSão Paulo, Brazil; ^5^Facultad de Ciencias Sociales, Universidad De PalermoBuenos Aires, Argentina; ^6^Department of Applied Psychology, School of Psychology, University of MinhoBraga, Portugal; ^7^Estudios Sobre Equidad y Genero and FES-Iztacala, Unidad de Investigación Interdisciplinaria en Ciencias de la Salud y la Educación, Proyecto Aprendizaje Humano, Centro Regional de Investigaciones Multidisciplinarias, Universidad Nacional Autonoma de MéxicoCuevarnaca, Mexico; ^8^School of Psychology, Victoria University of WellingtonWellington, New Zealand; ^9^Institute of Mental Health, Semmelweis UniversityBudapest, Hungary; ^10^Department of Psychology, University of OsloOslo, Norway; ^11^Department of Psychology, Claremont Graduate UniversityClaremont, CA, USA; ^12^Department of Humanities and Social Sciences, Indian Institute of Technology DelhiNew Delhi, India; ^13^Anugraha Institute of Social Sciences, Madurai Kamaraj UniversityDindigul, India

**Keywords:** happiness, lay definitions, adulthood, culture, inner harmony, relationships, interconnectedness

## Abstract

In well-being research the term happiness is often used as synonymous with life satisfaction. However, little is known about lay people's understanding of happiness. Building on the available literature, this study explored lay definitions of happiness across nations and cultural dimensions, analyzing their components and relationship with participants' demographic features. Participants were 2799 adults (age range = 30–60, 50% women) living in urban areas of Argentina, Brazil, Croatia, Hungary, India, Italy, Mexico, New Zealand, Norway, Portugal, South Africa, and United States. They completed the Eudaimonic and Hedonic Happiness Investigation (EHHI), reporting, among other information, their own definition of happiness. Answers comprised definitions referring to a broad range of life domains, covering both the contextual-social sphere and the psychological sphere. Across countries and with little variation by age and gender, inner harmony predominated among psychological definitions, and family and social relationships among contextual definitions. Whereas relationships are widely acknowledged as basic happiness components, inner harmony is substantially neglected. Nevertheless, its cross-national primacy, together with relations, is consistent with the view of an ontological interconnectedness characterizing living systems, shared by several conceptual frameworks across disciplines and cultures. At the methodological level, these findings suggest the potential of a bottom-up, mixed method approach to contextualize psychological dimensions within culture and lay understanding.

## Introduction

One of the most controversial issues in well-being research is the definition, investigation, and translation of the term “happiness.” Uchida and Ogihara (2012) highlighted considerable cultural differences in how lay people understand happiness, its predictors and its relation with social changes. However, only few researchers have empirically explored this still open question (Chiasson et al., 1996; Pflug, 2009; Delle Fave et al., 2011a). Moreover, these studies are not homogeneous, especially as concerns the formulation of the question used to investigate happiness definitions. In some studies participants are invited to define happiness *per se*, through questions like “what is happiness for you?” Other studies instead explore the perceived sources of happiness through questions like “what makes you happy?,” thus leaving happiness itself undefined or taking its meaning for granted. This difference, often overlooked, poses specific caveats in the interpretation of findings. Overall, sources of happiness were more frequently investigated than happiness *per se* (see for example Chiasson et al., 1996; Kim et al., 2007; Sotgiu et al., 2011), but they were often imprecisely described as “happiness definitions.” Moreover, studies on this topic prominently involve college students, representing a narrow specific age cohort and social class (e.g., Lu, 2001; Pflug, 2009).

Even less numerous works address linguistic and semantic features of the term “happiness” (Wierzbicka, 2009; Oishi et al., 2013). As discussed by Oishi and his colleagues, in many languages, including the Germanic family from which English stems, happiness is linguistically and conceptually related to fortune, positive fate, and luck. However, the meaning of the term gradually shifted toward a positive inner state, deriving from goal achievement and fulfillment of aspirations, especially in the US Protestant context. Other studies (Delle Fave et al., 2013) highlighted that in neo-Latin languages the term stems from the Latin “*felicitas*,” whose Indo-European root “fe” refers to growth, fertility and prosperity, thus, describing a developmental process rather than an achievement.

Presently, across social sciences “happiness” is predominantly used as synonymous with life satisfaction or Subjective Well-Being (SWB, Kahneman et al., 1999), a composite construct including the cognitive component of life satisfaction and the affective component of positive emotions (Veenhoven, 2012; Diener et al., 2013). Many instruments developed to assess happiness, and often used in studies conducted by social psychologists, sociologists, and economists, reflect this widespread approach (Burger et al., 2015). The traditional semantic use of the English term “happiness” (Oishi et al., 2013) fits the equation Happiness = Satisfaction, thus supporting its implementation in measurement. The term “happiness” is also imprecisely used to describe data collected through Cantril's ladder (Kilpatrick and Cantril, 1960). This single-item instrument invites participants to rate on a 0–10 scale how much their life is close to the “best possible life,” a condition usually treated in scientific studies as synonymous with happiness.

In the domain of psychology, within the so-called eudaimonic view other scholars have proposed different conceptualizations of happiness (Huta and Ryan, 2010; Huta and Waterman, 2014); however, these conceptualizations are more often subsumed under the umbrella term “well-being.” For instance, Psychological Well-Being (PWB; Ryff, 1989) comprises autonomy, positive relations, environmental mastery, self-acceptance, purpose in life, and personal growth. Eudaimonic Well-Being (EWB; Waterman, 2008) refers to self-expressiveness, development of inner potentials, and self-actualization.

Such a heterogeneous use of a single term is conducive to conceptual confusion. Despite the empirical evidence of positive correlations between life satisfaction, the best possible life, and happiness as a positive emotion (Rojas and Veenhoven, 2013), this conceptual ambiguity undermines the credibility of the happiness research domain. Problems arise especially when contradictory findings emerge, such as the high levels of happiness (used as synonymous with life satisfaction) shared by citizens of affluent, democratic and egalitarian countries such as Denmark, and citizens of Latin American countries, characterized by lower Gross Domestic Product, political instability and social insecurity (Rojas, 2006, 2012).

### Happiness studies across countries

Cross-country differences and similarities in the evaluation of happiness represent a still underexplored issue. Cultural awareness is increasingly acknowledged in the social sciences as an important resource for scientists and policy makers, promoting the respect for diversity, and preventing the unwitting imposition of values and concepts of one society on others (Christopher et al., 2014). In the last two decades some cultural dimensions that may influence happiness conceptualizations have been postulated or empirically identified (Uchida et al., 2004; Oishi et al., 2008; Joshanloo, 2014; Ramakrishna Rao, 2014).

The most frequently considered cultural dimension, that may influence happiness definitions, is Hofstede's (1980) construct of individualism/collectivism (I/C), prominently used in the comparison of Western and East Asian contexts (Oyserman et al., 2002; Uchida et al., 2004; Ford et al., 2015). Subsequent studies have provided a more fine-grained view, highlighting multiple types of collectivism across world regions, with different implications for psychological functioning (Ruby et al., 2012). Some of these differences may be explained in terms of another cultural dimension, defined as restraint vs. indulgence (Hofstede et al., 2010), and based on the extent to which people enjoy freedom of expression and personal control within their society. Another more recent conceptualization of value systems that may play a role in happiness definitions is that of Welzel and Inglehart (2010) who proposed the Inglehart-Welzel Cultural Map of the World, based on two categories: Traditional vs. Secular-rational values (according to the centrality attributed to religion, traditional family structure, deference to authority and national pride), and Survival vs. Self-expression values (according to the emphasis on economic and physical security vs. self-expression, subjective well-being, and interpersonal trust). These categories resonate in several respects with the two dimensions previously identified by Schwartz's (1992, 2012) theory of human values: openness to change vs. conservation, and self-enhancement vs. self-transcendence. This paper will focus on the conceptualizations developed within the first two models mentioned.

Particularly productive is the cross-cultural research around positive emotions, the affective dimension of happiness. While in Western countries happiness is characterized by an exclusively positive emotional valence, in East Asian ones it is often associated with a mixture of positive and negative emotions (Uchida, 2011). In the individualistic United States positive emotions are linked to the ideals of independence and personal achievement (Kitayama et al., 2006), while in collectivistic countries emotions connected to relations, such as interpersonal engagement, are predominant (Kitayama et al., 2000; Ford et al., 2015). Besides valence (positive vs. negative), researchers have explored the emotional dimension of arousal (Yik and Russell, 2003). Participants living in individualistic North American countries more often identify happiness with high arousal positive affect (HAP: excitement, euphoria, enthusiasm), while low arousal positive affect (LAP: serenity, peacefulness, tranquility) is preferred by collectivistic Eastern Asians (Lee et al., 2013). Further studies have highlighted more fine-grained variations across collectivistic countries: Mexicans and African groups tend to praise HAP in contrast to East Asians, who prefer LAP (Wissing and Temane, 2008; Ruby et al., 2012). The difference between these two typologies of collectivistic cultures may be linked to their opposite orientation on Hofstede's dimension of indulgence (predominant among Mexicans and Africans) vs. restraint (predominant among Asians) and position along Inglehart-Welzel's continuum of secular/rationale thinking (predominant among East Asians) vs. traditional thinking (typical of Latin Americans and Africans). Moreover, in individualistic countries older participants report LAP as the ideal mood more frequently than younger ones, suggesting age-related variations within the same culture (Tsai et al., 2006; Mogilner et al., 2011).

From the methodological point of view, most studies on happiness are characterized by a quantitative orientation. This represents a major challenge as concerns the possibility to capture cultural diversity (Hardin et al., 2014). Most instruments consist in scales, developed a priori by researchers trained in academic contexts, and thus possibly biased toward western individualistic notions of happiness (Uchida et al., 2004; Mathews, 2012). Moreover, these instruments do not provide information on lay people's view of happiness. As a first attempt to fill this gap, a cross-national study was conducted among adults living in seven Western countries, five of them within Europe. The primary aim of the study was to explore lay definitions of happiness through open-ended questions (the Eudaimonic and Hedonic Happiness Investigation—EHHI, Delle Fave et al., 2011a). Overall, the study allowed for distinguishing between definitions of happiness referring to life contexts and domains (conceptually related to the sources of happiness identified by the question “what makes you happy?”) and definitions referring to happiness as an inner state or dimension. Across nations, the most frequent contextual definitions of happiness were interpersonal relationships at both the family and broader social levels, while the most frequent psychological definition was inner harmony, an overarching dimension subsuming components such as emotional stability, LAP feelings of serenity and contentment, inner peace, acceptance, balance, and equipoise. The latter finding was surprising, especially because participants belonged to Western countries, while harmony (though conceptualized as a social dimension) is usually deemed as important in collectivistic cultures, primarily East Asian ones from which most studies on this topic were conducted (Ho and Chan, 2009; Ip, 2014; Sawaumi et al., 2015).

A more recent quantitative study conducted in a Western individualistic context confirmed the potential of inner harmony as a conceptualization of authentic-durable happiness, in contrast with fluctuating happiness (Dambrun et al., 2012). While the former includes the dimensions of inner peace and contentment (components of the “harmony” category in the EHHI study), the latter comprises high arousal emotions, pleasures, and the transient satisfaction related to achievements. From a similar perspective, a theoretical paper (Kjell, 2011) described inner harmony as an expression of sustainable well-being, discussing the heuristic potential of focusing on LAP and balance, rather than HAP and achievement.

Altogether, these studies suggested the need for delving more deeply into the individual understanding of happiness across cultures, investigating the extent to which happiness definitions provided by lay people dovetail with the definitions reported in their own language dictionaries, and with their countries' cultural features. It was necessary to further explore this issue in a larger sample of countries, including participants from different continents, and from both Western and Non-western contexts. It was also necessary to better understand the role of socio-demographic variables and country membership in the definitions of happiness, taking into account cultural dimensions and values formalized by current theories and models. Based on these premises, and adopting a bottom-up approach, the present study will explore lay conceptualizations of happiness through the collection of free definitions elicited by an open-ended question among adults from various countries. As an extension of the first EHHI study, data were gathered across a wider range of nations, and among participants from a larger age range.

### The present study

The aims of the study were: (1) to explore the psychological and contextual definitions of happiness reported by an international sample of adults in the productive life stage; (2) to investigate the relationship between happiness definitions and demographic features such as age, gender and education; (3) to explore the relationship between happiness definitions on the one hand, and country membership as well as cultural dimensions on the other hand.

Due to the exploratory nature of the study and the qualitative typology of the answers, we did not formulate specific hypotheses, but developed some guiding expectations based on the available empirical evidence. As concerns the first aim, inner harmony was expected to predominate among the psychological definitions of happiness across countries, in contrast with a low frequency of answers referring to luck and fortune. Family and social relationships were expected to represent the most frequent contextual definitions. As concerns the second aim, differences in happiness definitions were expected according to participants' age and marital status. More specifically parents, people married or cohabiting, and older participants were expected to provide a higher percentage of answers referring to family. As concerns the third aim of the study, we expected to identify differences related to the countries' scores in the cultural dimensions identified by Hofstede's model and Inglehart-Welzel's map. More specifically, we expected that people belonging to collectivistic countries would put more emphasis on relationships and social connections in their contextual definitions of happiness, compared with participants belonging to individualistic countries. In addition, the latter were expected to provide psychological definitions of happiness centered on life satisfaction and positive emotions more frequently than the former, who were instead expected to refer more frequently to harmony and balance. Based on the countries' position along the two dimensions of the Inglehart-Welzel's Map, participants from countries endorsing traditional values were expected to emphasize religion and relations (at the family, community and interpersonal levels) in the contextual definitions, and harmony in the psychological ones. In contrast, participants from countries more focused on secular and/or self-expressive values were expected to show a more pragmatic orientation toward work and leisure in the contextual definitions, and a greater emphasis on satisfaction and autonomy in the psychological ones. The discussion section will also include an effort to conceptually relate the study results to the happiness definitions provided by the local dictionaries of the examined countries.

## Materials and methods

### Countries and participants

The overall sample consisted of 2799 adults living in urban areas of 12 countries across different continents: Croatia, Italy, Hungary, Norway and Portugal in Europe; Argentina, Brazil, Mexico and United States in the Americas; India in Asia; South Africa in Africa; and New Zealand in Oceania. The countries' selection was based on researchers' professional contacts with colleagues from local Universities who were interested in the topics addressed by the study. This approach represents one of the three major strategies to conduct cross-cultural studies (Shiraev and Levy, 2010). A specific effort was made to include countries differing in geographic location and cultural traditions. Only four of the examined countries had been included in the previous EHHI study.

Table 1 reports the scores characterizing each of the examined countries on two dimensions identified by Hofstede (I/C and restraint/indulgence) and on the Inglehart-Welzel Map dimensions (4th and 5th wave of the World Value Survey). The 12 countries are widely distributed along the I/C continuum, with Portugal being the most collectivistic country and United States the most individualistic one. Ample variations can be detected also for the other cultural dimensions considered. Mexico hits the highest score for indulgence, followed by the English-speaking and Latin American samples, while India and the European countries (except for Norway) fall on the restraint side. As concerns the scores on Inglehart-Welzel dimensions, traditional values are endorsed by South Africa, followed by Portugal and Brazil, United States and the other Latin American countries, while secular values distinctively characterize Norway. Only Hungary scores high in survival values, while most of the other countries—especially Norway and the United States—endorse self-expressive ones. These profiles provide support to the substantial diversification of the samples under examination along the considered dimensions. In light of India's cultural, linguistic, and ecological diversity, two samples were included from this country, one from the northern state of New Delhi and Haryana (with Hindi as the official language) and the other one from the southern state of Tamil Nadu (whose official language is Tamil). Northern participants live in the metropolitan area of New Delhi, exposed to stronger modernization and secularization trends compared to the smaller and more traditional urban areas of Tamil Nadu. Differences between the two states are grounded in history, since during the last millennium North India underwent repeated foreign invasions from Asian and European populations that contributed to shape the complex mixture of languages, customs, religions and values presently characterizing the region. The southern-eastern region was instead relatively immune to cultural contaminations. Community ties were preserved across the centuries within a substantially peaceful and stable social environment (Kulke and Rothermund, 1990).

**Table 1:** **Scores of the examined countries on two Hofstede's dimensions and Inglehart-Welzel's Map dimensions**.

**Country**	**Individualism/collectivism**	**Indulgence/restraint**	**Traditional/Secular values**	**Survival/Self expressive values**
United States	91	68	−0.81	1.76
New Zealand	79	75	0.00	1.86
Italy	76	30	0.13	0.60
Norway	69	55	1.39	2.17
South Africa	65	63	−1.09	−0.10
Hungary	55	31	0.40	−1.22
India	48	26	−0.36	−0.21
Argentina	46	62	−0.66	0.38
Brazil	38	59	−0.98	0.61
Croatia	33	33	0.08	0.31
Mexico	30	97	−1.47	1.03
Portugal	27	33	−0.90	0.49

*Countries are rank ordered for Individualism*.

Each country contributed to the global sample with 216 participants, except for New Zealand (215) and Argentina (208). Each local sample was balanced by age, gender and education level. Participants' age ranged between 30 and 60 (mean age 44.2). Each local sample included 108 men and 108 women, equally distributed across three age ranges (covering 10 years each) and two levels of education (high school and college). Most participants (93.3%) had a full-time job, prominently office work (24.30%), helping professions (22.62%), business, and private entrepreneurship (20.64%), unspecialized work (13.1), and science and technology (9.13%). The majority was married or cohabiting with a partner (72.9%), 16.3% were single and 10.8% were separated, divorced or widowed. Most participants (80.7%) had children. Over half of the interviewees (56.7%) were Christian, 12.4% were Hindu, and 27.6% reported being atheist/not belonging to a religion. These demographic features were consistent with the aim of investigating happiness notions among adults who had experienced major life transitions in education, work, and family.

### Materials

Participants were administered the Eudaimonic and Hedonic Happiness Investigation (EHHI; Delle Fave et al., 2011a), that investigates various dimensions of well-being through Likert scales and open-ended questions. In this paper, we will focus on the open-ended question inviting participants to define happiness in their own words: “What is happiness for you?” After completion of the EHHI, participants were asked to fill out a Socio-Demographic Questionnaire providing information on their gender, age, level of education, employment status, occupation, family structure, and religion. In each country, the instruments were administered in the local language, and the term “happiness” was translated into the word most commonly used in daily language. The terms used for translations and their definitions in the national dictionaries are reported in Appendix A in Supplementary Material. In Northern India, based on the daily use of words belonging to two local languages—Urdu and Hindi—the question formulation included two terms.

As shown in Appendix A in Supplementary Material, the dictionary definitions of happiness in the examined countries prominently refer to positive emotions and feelings (joy, cheerfulness, enthusiasm, pleasure, contentment). When these feelings arise in relation to positive outcomes or achievements based on individual effort and agency, happiness is described through the evaluative term of satisfaction. In the majority of countries, except for Hungary, Italy, Mexico, and North India, dictionary definitions also include “luck” or “fortune,” though in the US dictionary this definition is labeled as “obsolete.” Oishi et al. (2013) highlighted that this interpretation became gradually less popular in the Anglo-Saxon context, and it disappeared from the most recent dictionary editions; according to these authors, the nations in which it is still common usually report lower levels of happiness.

### Procedure

A project coordinating committee was identified, comprising researchers who had conducted previous EHHI studies. The committee drew up guidelines and procedural rules, and local researchers implemented the study in each country. Approval from the ethics committees of the researchers' institutions and written informed consent from participants were obtained according to local rules and legal provisions and in line with the Helsinki Declaration. Participation was voluntary in all instances. Local researchers recruited participants through face-to-face interaction in public areas, word-of-mouth, and non-probability sampling. Most participants filled out the questionnaire autonomously and returned it to the local researcher personally, by mail, email, or online (the latter option was only available in New Zealand and United States). The local researcher removed the informed consent from the answer sheets, and numbered them correspondingly. Questionnaires were thus, handled anonymously in coding and analyses. The coded responses were stored on password protected computers.

#### Coding procedure

Since most participants provided multifaceted descriptions of happiness, their answers were partitioned into smaller, semantically different units. Each unit was coded separately as one numeric item. Up to six answer units were retained for each participant. This decision was based on the assessment of the percentage of participants reporting the same number of answer units. More specifically, the majority of the participants (71.2%) provided 1 to 5 units, in the following proportion: five units 23.9%, four units 18.2%, three units 11.5%, two units 8.8%, and one unit 8.7%, while the remaining 28% provided 6 answer units. Within this last subgroup, only very few people in each country provided additional units, that were thus discarded from analyses. The coding procedure was performed using the coding system developed for the previous EHHI study (Delle Fave et al., 2011a). This coding system is organized in broad functional categories, further subsuming sub-categories. Single items are classified within this hierarchical system, as units of subcategories within broader categories.

The grouping of items within coding categories and subcategories was prominently data driven, in line with the bottom-up approach characterizing the study. At the same time, specific theoretical frameworks were taken as reference points. The identification of categories was oriented by the research line of quality of life studies, focusing on a variety of contextual-social and psychological domains (WHOQOL Group, 2004), whose perceived quality levels are analyzed within communities and populations. The EHHI answers could be grouped into the following broad categories: work, family, standard of living, interpersonal relations, health, leisure, spirituality/religion, society and community issues, education, and psychological states. As concerns the identification of subcategories, the answer contents allowed for grouping most subsets of items according to their relation with facets of well-being described in the scientific literature, whose definition was used as the label for the corresponding subcategories (Delle Fave et al., 2013). In particular, within the category of psychological definitions most items could be included into subcategories corresponding to constructs elaborated within the hedonic and eudaimonic conceptualizations of well-being (Huta and Waterman, 2014): satisfaction and positive emotions (components of SWB, Kahneman et al., 1999); autonomy, mastery, purpose, and personal growth (components of Ryff's PWB model); meaning in life (Steger et al., 2006); self-actualization (Waterman, 2008); and optimism (Peterson, 2000). Moreover, the data-driven, bottom-up approach to answer coding led to the addition of subcategories substantially neglected in studies on happiness definition: inner harmony (including low-arousal feelings of peacefulness, serenity, balance and equipoise), awareness, and absence of negative feelings (Delle Fave et al., 2011a). The categories of family, interpersonal relationships, and community/society were partitioned into the sub-categories of intrinsic value/meaning, personal contribution, sharing/reciprocity, well-being (of family, community, society members), and personal reward. Work comprises the sub-categories of engagement/competence, self-actualization/ expressiveness, intrinsic value/meaning, satisfaction/achievement, structural changes/improvements, harmony, social recognition, and standard of living. Religion/spirituality includes faith cultivation, spiritual growth, and religious practice.

The structure and organization of the EHHI coding systems allow for the inclusion of additional items derived from the progressive data collection through new studies, thus leading to an increasingly exhaustive mapping of lay people definitions of happiness across countries, life stages, gender, and education levels. This expansion of the coding system occurred for the present study as well. However, while new items were added to the existing categories and subcategories, it was not necessary to expand the number of categories and subcategories. The classification approach adopted thus, seems to be suited to the typology and contents of the answers provided by participants across countries (Delle Fave et al., 2013). In each country, two trained raters transformed each answer unit into a numeric item. Discrepancies were discussed until consensus was reached. Further doubts or disagreements were resolved through the involvement of two members of the coordinating committee, in order to ensure the trustworthiness of the coding. The updated codebook comprised 1511 items, of which 366 referred to happiness definitions. Among them, 149 were included in the psychological states category.

#### Statistical analyses

Descriptive statistics, logistic regressions, and correspondence analyses were performed separately for the different categories of happiness definitions. Descriptive findings represented the basis for subsequent inferential analyses. Standard binary logistic regressions were used to identify the demographic predictors (age, gender, education, marital status, religion, and country membership) and cultural predictors (based on the dimensions identified by Hofstede's and Inglehart-Welzel's models) of specific categories and subcategories of happiness definitions, which represented the dependent variables. The possibility to use multilevel regression analyses was considered, as this approach takes into account the hierarchical structure of our multi-country dataset. However, at least 30 countries are required to reliably estimate individual-level effects within each country in logit models (Bryan and Jenkins, 2015), and this condition was not satisfied by the present dataset.

As concerns the analyses involving country as demographic predictor, Portugal was identified as the reference country, based on the scores obtained on the four cultural dimensions considered, as well as geographical and historical reasons. Among the examined countries, Portugal lies on the collectivism and restraint pole of Hofstede's ranking, and toward the extreme of Inglehart-Welzel's traditional values dimension. It may thus, represent a well-characterized reference point to which to compare the other countries, more variably fluctuating across these dimensions. Moreover, Portugal represents a geographical bridge between Eurasia and Americas, as well as a cultural bridge between Europe and some of the countries included in the sample, based on its prolonged contact and influence on Latin American cultures (prominently Brazil, characterized by the same language and polarization toward collectivism and traditional values), South India (sharing with Portugal high scores on restraint) and Africa during the colonial era. Compared to other criteria that could be adopted to identify a reference country, such as indicators of socio-economic development, this approach was deemed as more pertinent to the major study aim, namely the exploration of well-being in relation to lay people's understanding and language use.

Finally, correspondence analyses were used to explore the relationships between country membership and categories/subcategories of happiness definitions. This exploratory technique for categorical data aims to find a minimum number of dimensions to account for the maximum amount of inertia (analogous to the total variance in principal component analysis). It defines a measure of distance between any two points (categories) in terms of the distances between individual rows or columns in a low-dimensional space. Principal component analysis, performed on the distance matrix, yields the dimensions that are used to map points. Each row and column of the table becomes a point on a graphical map, which typically consists of two or three dimensions. Since correspondence analysis is an exploratory technique for interpreting the data, statistical significance of relationships should not be assumed (Greenacre, 2007).

## Results

### Descriptive findings

#### Contextual and psychological categories of happiness definitions

**Figure 1:**
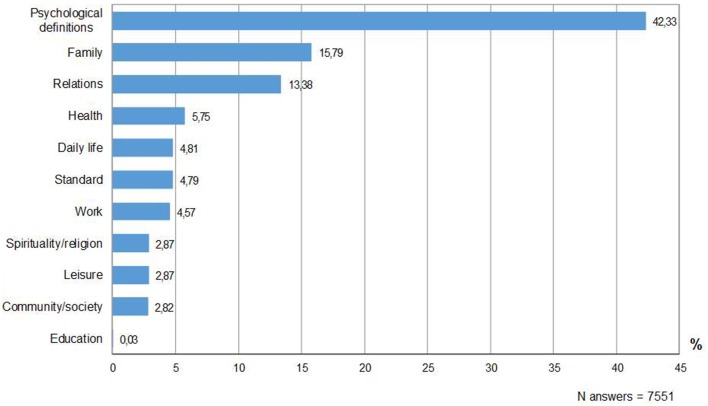
**Happiness definitions: percentage distribution of answers across categories**.

Overall, participants provided 7551 definitions of happiness. Thirty-five participants (1.25%) did not provide any answer, and 48 (1.74%) stated that “happiness does not exist.” The percentage distribution of happiness definitions, grouped into categories, is presented in Figure 1. Psychological definitions represented the most frequent category. They included descriptions of happiness as an inner state, feeling or attitude. All the other categories referred to specific life domain and contexts, and they were globally grouped under the label of of “contextual definitions.” Overall and in 11 of the 12 nations, the most frequently mentioned contextual categories were family and interpersonal relationships, followed by health, daily life, standard of living, and work. As reported in the procedure section, the articulation of categories into subcategories allowed for a more fine-grained inspection of the findings. Within the category “family,” the most frequent subcategories were sharing (happiness as solidarity, cohesion and mutual support, accounting for the 44.46% of the answers), well-being (mental and physical health of family members, children's positive growth, goal attainment in the family, 24.83% of the answers), and intrinsic value (happiness as the presence of family, children, partner in one's own life; 18.20%). Within the category “interpersonal relations,” definitions mainly referred to the intrinsic value of having friends and significant others (26.73% of the answers), sharing life experiences (25.54%), providing a positive contribution to others (19.41) and getting support, respect and recognition from them (18.42%).

**Figure 2:**
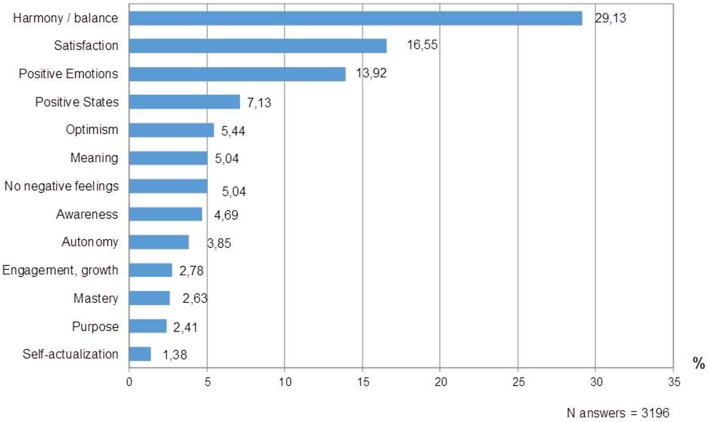
**Psychological definitions: percentage distribution of answers across subcategories**.

In light of the high frequency of psychological definitions of happiness, and in line with the related literature (in which happiness is conceptualized as a psychological state), specific attention was devoted to the analysis of this category. Figure 2 shows the distribution of the psychological definitions of happiness. Harmony, accounting for almost 30% of the psychological answers, was the subcategory mentioned most frequently, overall and in 11 of the 12 countries. Four components were identified in this subcategory. Inner peace (37.06% of the answers) included peace of mind, emotional stability, detachment, tranquility, and serenity; balance (29.11%), comprised feelings of inner balance, inner harmony, acceptance of life, being attuned with the universe, and balance between wishes and achievements; contentment (23.20%), comprised contentment in general and with oneself; and psychophysical well-being (10.63%) represented a single-item component, without further specifications. Satisfaction and positive emotions followed in rank. Satisfaction included answers referring to attainment of life goals, realization of dreams and expectations, satisfaction with life and oneself. Positive emotions prominently included HAP feelings such as joy, cheerfulness, vitality, enthusiasm, and elation (71% of the answers in this sub-category), and with lower percentages LAP feelings such as pleasure and comfort (29%). Positive states ranked fourth, prominently referring to a general “state of well-being,” “mental well-being,” and in marginal percentages, specific experiences such as flow, absorption in a task, performing activities without self-consciousness. Optimism, meaning, absence of negative feelings, and awareness (of oneself as a person; of the present moment) followed in rank. Finally, engagement/growth, purpose, mastery, and autonomy accounted for less than 4% of the psychological answers each.

#### Definitions of happiness across countries

The percentage of participants by country who provided at least one answer for each category of happiness definition is illustrated in Table 2. The chi square procedure was adopted to compare the distribution of participants in each category across countries. For each category, a contingency table was produced including 13 samples × 2 answer options (0, no answer in the category; 1, at least one answer in the category). These separate contingency tables are summarized in Table 2, which presents the percentage of participants who quoted each happiness definition category (13 samples × 10 categories). For sake of synthesis, only the cells referring to the positive option (at least 1 answer in the category) are reported in the Table. Given the large sample size, a conservative approach was adopted to investigate differences in the percentage of participants mentioning each category across countries. Instead of relying on cell chi-square values, adjusted residuals were calculated for each cell. Only values above 3.29 (*p* < 0.001) were considered as significant, and reported in brackets below the percentage of participants. Due to the large amount of data, the description of results will focus on the major findings.

**Table 2:** **Definitions of happiness: percentage of participants who mentioned each category by country**.

	**Portugal**	**Italy**	**Norway**	**Croatia**	**Hungary**	**USA**	**Mexico**	**Argentina**	**Brazil**	**S.Africa**	**N.India**	**S.India**	**N.Zealand**	**χ^2^**
Psychological definitions	85.65 (6.1^*^)	87.04 (6.5^*^)	58.33	46.30 (−6.7^*^)	59.26	53.70 (−4.3^*^)	62.04	76.92	70.37	75.46	48.61 (−6.0^*^)	61.57	85.12 (5.9^*^)	232.83^**^
Family	22.22 (−3.3^*^)	20.37 (−4.0^*^)	61.11 (9.4^*^)	40.28	47.69 (5.0^*^)	48.61 (5.3^*^)	18.06 (−4.7^*^)	10.10 (−7.2^*^)	33.80	21.76 (−3.5^*^)	29.17	37.50	30.7	243.33^**^
Interpersonal relations	46.3 (5.8^*^)	29.63	53.7 (8.3^*^)	31.02	28.24	28.24	13.43 (−5.3^*^)	6.25 (−7.5^*^)	31.48	28.70	10.19 (−6.4^*^)	23.15	46.51 (5.9^*^)	246.89^**^
Health	17.59	11.57	26.39 (4.7^*^)	26.39 (4.7^*^)	24.07 (3.7^*^)	13.43	6.02 (−4.0^*^)	4.81 (−4.4^*^)	22.22	9.72	14.35	12.50	10.23	108.14^**^
Work	19.44 (3.5^*^)	5.09	28.24 (7.7^*^)	18.06	16.2	12.50	1.85 (−4.8^*^)	0.96 (−5.1^*^)	18.98 (3.3^*^)	9.26	9.26	2.31 (−4.5^*^)	12.56	164.35^**^
Standard of living	8.800	2.78 (−4.3^*^)	26.39 (6.8^*^)	14.81	23.61 (5.5^*^)	10.19	7.87	2.4 (−4^*^)	7.87	10.19	13.43	6.48	20.47 (4.0^*^)	139.05^**^
Leisure	2.78	1.39	24.07 (11.8^*^)	6.94	3.70	11.57 (3.7^*^)	0.46 (−3.5^*^)	0 (−3.8^*^)	3.24	2.78	0 (−3.8^*^)	1.85	18.14 (7.9^*^)	262.77^**^
Spirituality, religion	9.26	4.17	1.85	5.56	3.70	10.65	5.09	4.33	11.11	12.50 (3.3^*^)	1.39 (−3.4^*^)	15.74 (5.2^*^)	6.05	76.70^**^
Community, society	9.26	6.48	5.56	5.09	3.70	7.87	0.93 (−3.7^*^)	0.96 (−3.6^*^)	3.24	2.31	12.04	22.22 (9.0^*^)	12.09	135.92^**^
Daily life	10.19	8.80	14.35	17.59	10.65	9.26	6.02	11.06	19.44 (3.5^*^)	17.59	6.48	7.41	16.74	52.68^**^
N participants^a^	216	216	216	216	216	216	216	208	216	216	216	216	215	

a *Each participant could provide more than one answer; Values in brackets represent significant adjusted residuals: Cut-off value = 3.29*;

* *p < 0.001*;

** *p < 0.0001*.

Psychological definitions were reported by a significantly higher percentage of participants from Italy, Portugal, and New Zealand, in contrast with the significantly lower percentage of participants referring to them in the United States, North India and Croatia. A significantly higher percentage of participants from Norway, Hungary, and United States provided happiness definitions within the domain of family, while the opposite trend was detected in Argentina, Mexico, Italy, and Portugal. Relations were mentioned by significantly higher percentages of participants form Norway, New Zealand, and Portugal, and by lower percentages of Argentineans, North Indians, and Mexicans. The domain of health was cited by significantly higher percentages of participants from Croatia, Norway and Hungary, and work by Portuguese and Norwegians. Leisure and standard of living were reported by a significantly higher percentage of participants from New Zealand and Norway. Leisure was also prominent among US participants, and standard of living among Hungarians. Only South Indians referred in significantly higher percentage to community and society and, together with South Africans, to spirituality and religion.

As previously stated, the conceptual and empirical predominance of psychological definitions implied the need for a closer investigation of the findings included in this category. Table 3 shows the percentage distribution of participants reporting at least one answer across countries for the major subcategories of psychological definitions (subcategories cited by less than 10% of the participants across countries are not included). Harmony was cited by the highest percentage of participants in all countries but Croatia. The percentage of participants referring to harmony was significantly higher in Italy, Hungary and South India, and significantly lower in Mexico. Positive emotions and satisfaction followed in rank across countries, with the exception of Brazil and North India that included a higher percentage of participants defining happiness as a positive state. Some subcategories were reported by more than 10% of the participants in only few samples: meaning in South Africa, New Zealand and South India, awareness in Italy and Argentina, autonomy in South Africa and Norway, and mastery in Norway. Specific attention deserves the subcategory “no negative feelings,” prominently reported by participants from New Zealand, United States, Croatia, and Norway; it represents the only case in which happiness is described as the absence of problems rather than presence of positive indicators.

**Table 3:** **Psychological definitions of happiness: percentage of participants who mentioned each subcategory by country**.

	**Portugal**	**Italy**	**Norway**	**Croatia**	**Hungary**	**USA**	**Mexico**	**Argentina**	**Brazil**	**S.Africa**	**N.India**	**S.India**	**N.Zealand**	**χ^2^**
Harmony	43.78	55.85 (4.3^*^)	31.75	25.00 (−3.4^*^)	57.03 (3.8^*^)	42.24	26.12 (−3.7^*^)	41.88	30.26	34.97	37.14	56.39 (3.7^*^)	42.62	82.25^**^
Satisfaction	35.68 (3.5^*^)	27.13	7.94 (−4.6^*^)	48.00 (5.4^*^)	22.66	27.59	21.64	16.25	25.66	26.99	18.10	21.05	26.78	54.42^**^
Positive emotions	28.11	13.30	24.60	15.00	15.63	19.83	17.91	19.38	22.37	33.13 (3.8^*^)	10.48	17.29	32.24 (3.7^*^)	71.88^**^
Positive states	21.62 (4.3^*^)	7.98	11.11	1.00 (−3.5^*^)	4.69	8.62	7.46	0 (−4.9^*^)	28.29 (6.5^*^)	16.56	14.29	15.04	11.48	107.05^**^
No negative feelings	6.49	6.38	14.29	11.00	5.47	15.52	1.49	1.88	3.29	9.82	8.57	4.51	14.75 (3.7^*^)	55.21*
Meaning	9.19	6.38	10.32	10.00	4.69	8.62	2.99	0-(3.8^*^)	3.29	12.88	5.71	15.04 (3.3^*^)	10.93	46.03^**^
Awareness	5.95	10.64	6.35	9.00	4.69	6.03	6.72	18.13 (5.2^*^)	3.95	9.82	4.76	6.02	5.46	37.57^**^
Optimism	2.16 (−3.3^*^)	13.83	8.73	9.00	10.16	14.66	6.72	5.00	7.89	11.66	3.81	1.50	13.66	45.45^**^
Autonomy	2.16	3.72	12.70 (3.6^*^)	11.00	4.69	2.59	1.49	3.75	4.61	12.88 (4.2^*^)	1.90	1.50	9.84	60.14^**^
Mastery	5.95	4.26	14.29 (5.6^*^)	2.00	2.34	5.17	0.75	0	0.66	6.75	0	6.02	7.65	61.90^**^
N participants^a^	185	188	126	100	128	116	134	160	152	163	105	133	183	

a *Each participant could provide more than one answer; Values in brackets represent significant adjusted residuals: Cut-off value = 3.29*;

* *p < 0.001*;

** *p < 0.0001*.

Given the primacy of harmony among the psychological definitions of happiness, the components of this subcategory were analyzed in detail across countries, and related findings are presented in Table 4. Inner peace emerged as the most frequent component, followed by inner balance. Looking at the participants' distribution for each component, Italians referred to inner peace in a significantly higher percentage. Inner balance predominated in Norway and Hungary, while a significantly lower percentage of Indian participants referred to it. Contentment yielded a significantly higher percentage of participants from South India, Portugal, USA, South Africa, and New Zealand, while it was reported by a significantly lower percentage of Italians, Hungarians, Argentineans, and Brazilians.

**Table 4:** **Inner harmony: percentage of participants who mentioned each component by country**.

	**Portugal**	**Italy**	**Norway**	**Croatia**	**Hungary**	**USA**	**Mexico**	**Argentina**	**Brazil**	**S.Africa**	**N.India**	**S.India**	**N.Zealand**	**χ^2^**
Inner peace	24.69	60.00 (4.2^*^)	22.50	48.00	38.36	40.82	40.00	32.84	54.35	36.84	61.54	52.00	25.64	54.69^**^
Balance	20.99	42.86	70.00 (5.3^*^)	32.00	49.32 (3.3^*^)	20.41	31.43	44.78	36.96	17.54	7.69 (−3.3^*^)	8.00 (−4.7^*^)	32.05	91.38^**^
Contentment	53.09 (5.4^*^)	6.67 (−5.2^*^)	7.50	0.00	4.11 (−4.7^*^)	55.10 (4.4^*^)	22.86	8.96 (−3.6^*^)	4.35 (−3.6^*^)	50.88 (4.1^*^)	15.38	58.67 (6.3^*^)	44.87 (3.6^*^)	196.14^**^
Psychophysical WB	16.05	9.52	15.00	24.00	20.55	0.00	8.57	19.40	17.39	10.53	17.95	0.00 (−3.5^*^)	15.38	32.46*
N participants^a^	81	105	40	25	73	49	35	67	46	57	39	75	78	

a *Each participant could provide more than one answer. Values in brackets represent significant adjusted residuals: Cut-off value = 3.29*;

* *p < 0.001*;

** *p < 0.0001*.

### Relationship between socio-cultural features and happiness definition categories

#### Demographic predictors

Binary logistic regressions were carried out to investigate which demographic features predicted the mention of each happiness definition category. Related findings are presented in Table 5. Dependent variables were work, family, standard of living, interpersonal relations, health, psychological definitions, spirituality, and community/society issues. Leisure was discarded based on the low percentage of mentions (less than 4% of the participants referred to it in 9 of the 13 samples), and life in general due to the lack of specificity. Demographic variables (age, gender, marital status, belonging to a religion, and country) were used as predictors. All logistic models significantly improved prediction when compared to baseline models. The amount of explained variance of the dependent variables varied from 8% (work, health, and community/society issues) to 16–17% (family and psychological definitions).

**Table 5:** **Binary logistic regression: demographic and country predictors of happiness definition categories**.

**Predictors**	**Work^b^**			**Family**			**Standard of living**			**Interpersonal relations**		
	**B**	**SE**	**OR**	**B**	**SE**	**OR**	**B**	**SE**	**OR**	**B**	**SE**	**OR**
Age (30 years)^a^	0.01	0.01	1.01	0.00	0.01	1.00	0.00	0.01	1.00	0.00	0.01	1.00
**GENDER (FEMALE)^a^**												
Male	0.11	0.12	1.12	−0.24^*^	0.09	0.79	0.35^*^	0.12	1.42	−0.42^**^	0.09	0.65
Education (secondary)^a^												
Tertiary	0.09	0.12	1.09	−0.46^**^	0.09	0.63	−0.40^**^	0.12	0.67	0.19	0.09	1.21
**MARITAL STATUS (SINGLE)^a^**												
Married	−0.27	0.18	0.76	0.90^**^	0.14	0.90	0.06	0.19	1.06	−0.08	0.13	0.93
Cohabiting	0.02	0.24	0.98	0.68^**^	0.19	0.68	0.19	0.25	1.21	−0.09	0.18	0.92
Divorced/widowed	−0.33	0.26	0.72	0.62^**^	0.19	0.62	0.44	0.24	1.55	−0.09	0.18	0.91
**RELIGION (NONE)^a^**												
Having religion	0.25	0.17	1.29	0.15	0.13	0.15	0.34	0.17	1.41	−0.03	0.13	0.97
**COUNTRY (PORTUGAL)^a^**												
Italy	−1.57^**^	0.36	0.21	0.01	0.24	1.01	−1.20	0.48	0.30	−0.75^**^	0.21	0.47
Norway	0.49	0.24	1.64	1.88^**^	0.23	6.54	1.37^**^	0.30	3.92	0.28	0.20	1.32
Croatia	−0.05	0.25	0.95	0.85^**^	0.22	2.35	0.66	0.31	1.94	−0.68^**^	0.20	0.50
Hungary	−0.08	0.28	0.92	1.37^**^	0.24	3.93	1.42^**^	0.32	4.13	−0.83^**^	0.23	0.44
USA	−0.56	0.27	0.57	1.38^**^	0.22	3.97	0.22	0.33	1.24	−0.81^**^	0.21	0.44
Mexico	−	−	−	−0.20	0.24	0.82	−0.15	0.35	0.86	−1.75^**^	0.24	0.17
Argentina	−	−	−	−0.83^**^	0.29	0.44	−1.41^*^	0.51	0.24	−2.59^**^	0.32	0.07
Brazil	−0.05	0.25	0.95	0.69^**^	0.22	2.00	−0.12	0.35	0.88	−0.66^**^	0.20	0.52
South Africa	−0.90^**^	0.29	0.41	0.06	0.24	1.06	0.12	0.33	1.13	−0.76^**^	0.21	0.47
North India	−0.86^**^	0.29	0.42	0.32	0.23	1.38	0.50	0.31	1.65	−2.04^**^	0.27	0.13
South India	−2.33^**^	0.48	0.10	0.69^**^	0.22	1.98	−0.33	0.37	0.72	−1.07^**^	0.21	0.34
New Zealand	−0.42	0.28	0.65	0.58	0.23	1.78	1.14^**^	0.31	3.11	−0.02	0.21	0.98
Intercept	−1.80^**^	0.40	0.15	−1.08^**^	0.31	0.34	−2.72^**^	0.43	0.07	0.34	0.29	1.40
Nagelkerke R2 (%)	8.52			15.58			10.97			14.11		
**Predictors**	**Health**			**Psychological definitions**			**Religion/Spirituality^b^**			**Community and society issues^b^**		
	**B**	**SE**	**OR**	**B**	**SE**	**OR**	**B**	**SE**	**OR**	**B**	**SE**	**OR**
Age (30 years)^a^	0.02	0.01	1.02	−0.01	0.01	0.99	0.03^**^	0.01	1.03	0.02	0.01	1.02
**GENDER (FEMALE)^a^**												
Male	−0.16	0.11	0.85	−0.11	0.09	0.90	0.01	0.15	1.04	0.07	0.16	1.07
**EDUCATION (SECONDARY)^a^**												
Tertiary	−0.36^**^	0.11	0.70	0.82^**^	0.09	2.27	0.33	0.15	1.42	−0.06	0.15	0.94
**MARITAL STATUS (SINGLE)^a^**												
Married	0.06	0.17	1.07	−0.36^*^	0.13	0.70	0.35	0.25	1.42	−0.11	0.24	0.89
Cohabiting	0.07	0.23	1.07	0.08	0.19	1.08	0.09	0.45	1.09	−0.21	0.38	0.81
Divorced/widowed	0.19	0.22	1.21	−0.10	0.18	0.91	−0.16	0.36	0.85	−0.24	0.35	0.78
**RELIGION (NONE)^a^**												
Having religion	0.04	0.16	1.04	−0.10	0.13	0.90	2.19^**^	0.42	7.98	0.30	0.25	1.36
**COUNTRY (PORTUGAL)^a^**												
Italy	−0.48	0.28	0.62	0.01	0.29	1.01	−0.79	0.42	0.46	−0.40	0.37	0.67
Norway	0.51	0.25	1.66	−1.68^**^	0.25	0.19	−	−	−	−0.45	0.39	0.64
Croatia	0.56	0.24	1.75	−2.03^**^	0.25	0.13	−0.41	0.38	0.66	−0.59	0.39	0.55
Hungary	0.43	0.27	1.54	−1.63^**^	0.26	0.20	0.24	0.47	1.27	−0.76	0.46	0.47
USA	−0.31	0.27	0.73	−1.82^**^	0.25	0.16	0.42	0.33	1.52	−0.19	0.35	0.82
Mexico	−1.20^**^	0.34	0.30	−1.43^**^	0.25	0.24	−0.60	0.39	0.55	−	−	−
Argentina	−1.45^**^	0.37	0.23	−0.73^*^	0.26	0.48	−0.66	0.42	0.52	−	−	−
Brazil	0.29	0.24	1.34	−1.03^**^	0.25	0.36	0.29	0.32	1.33	−1.11	0.45	0.33
South Africa	−0.68	0.29	0.51	−0.74^*^	0.26	0.48	0.31	0.32	1.36	−1.48^*^	0.51	0.23
North India	−0.22	0.27	0.80	−1.93^**^	0.24	0.15	−	−	−	0.28	0.32	1.32
South India	−0.41	0.27	0.66	−1.34^**^	0.25	0.26	0.48	0.30	1.62	1.00^**^	0.29	2.71
New Zealand	−0.62	0.30	0.54	−0.18	0.29	0.83	0.29	0.39	1.34	0.46	0.34	1.58
Intercept	−1.14^**^	0.37	0.32	1.30^**^	0.32	3.67	−5.66^**^	0.65	0.00	−2.69^**^	0.53	0.07
Nagelkerke R2 (%)	8.16			16.78			10.86			8.56		

* *p < 0.05*;

** *p < 0.01 (Bonferroni correction); OR, Odds ratio*.

a *Reference category*.

b *Some countries were excluded from this analysis because less than five participants reported the specific category*.

Age emerged as a relevant predictor for spirituality/religion, more often mentioned by older participants. Women were more prone to refer to family and interpersonal relations, and men to mention standard of living. People with college education more often reported psychological dimensions, and less frequently family, standard of living, and health. Single participants were less likely to provide family related definitions, and married individuals to provide psychological definitions. Religious affiliation significantly predicted the likelihood of mentioning religion/spirituality.

In order to investigate country membership as predictor of specific definition categories, Portugal was taken as the reference country. Countries with less than five participants providing answers in a specific category were excluded from the related regression procedure. Significant country differences were found for most categories. Community/society issues and spirituality/religion showed smaller variations across countries, but they were reported by an overall limited number of participants. Larger differences were detected for interpersonal relations, psychological definitions, and family. Consistent with the descriptive findings, a significantly lower probability to quote interpersonal relations compared to Portugal was detected among participants from most countries, except Norway and New Zealand.

The probability of mentioning psychological definitions of happiness was significantly lower than in Portugal among participants from most countries, except New Zealanders and Italians. Participants from three European countries (Norway, Hungary and Croatia), two American ones (Brazil and US), and South India had a significantly higher probability to quote family than Portuguese participants, while the opposite trend emerged for Argentineans. Country was the only significant predictor for work-related definitions. In particular, compared to Portugal, living in India, Italy, and South Africa predicted a lower probability to do so. Argentinians and Mexicans were significantly less prone to define happiness as health than Portuguese participants. Compared to Portugal, participants from Norway and Hungary referred to standard of living in significantly higher percentages, while the opposite trend was detected for Argentineans. South Indians were more likely to refer to community and society than Portuguese, while South Africans were significantly less likely to do so.

#### Cultural predictors

Binary logistic regressions were also performed to investigate the role of Hofstede's and Inglehart-Welzel's cultural dimensions and values in predicting each happiness definition category. The four dimensions reported in Table 1 (individualism/collectivism, indulgence/restraint, traditional/secular values, and survival/self-expression values) were used as predictors. As shown in Table 6, the dimensions identified by Inglehart-Welzel's model were the prominent and strongest predictors of happiness definitions. In particular, participants from countries endorsing secular values were significantly more prone to refer to family and relationships, as well as work, standard of living and health, while participants from countries endorsing traditional values more frequently reported religion, and psychological definitions of happiness. Moreover, living in a country characterized by self-expression values predicted a higher tendency to report work, relationships, and community/society issues.

**Table 6:** **Binary logistic regression: cultural dimensions and values as predictors of happiness definition categories**.

**Predictors^a^**	**Work^1^**			**Family**			**Standard of living**			**Interpersonal relations**		
	**B**	**SE**	**OR**	**B**	**SE**	**OR**	**B**	**SE**	**OR**	**B**	**SE**	**OR**
Individualism/collectivism	−0.01	0.00	0.99	0.00	0.00	1.00	−0.00	0.00	1.00	−0.00	0.00	1.00
Indulgence/restraint	−0.01	0.01	0.99	0.00	0.00	1.00	0.02^*^	0.01	1.02	−0.01^**^	0.00	1.00
Traditional/secular values	0.39^**^	0.10	1.47	0.52^**^	0.07	1.68	0.80^**^	0.10	2.23	0.22^*^	0.08	1.25
Survival/expression values	0.33^**^	0.10	1.40	0.07	0.07	1.07	−0.24	0.09	0.79	0.45^**^	0.07	1.57
Intercept	−0.84^**^	0.24	0.43	−0.83^**^	0.18	0.44	−2.27^**^	0.27	0.10	−0.43	0.17	0.65
Nagelkerke R2 (%)	1.16			5.59			4.79			5.11		
**Predictors**	**Health**			**Psychological definitions**			**Religion/Spirituality^2^**			**Community and society issues^1^**		
	**B**	**SE**	**OR**	**B**	**SE**	**OR**	**B**	**SE**	**OR**	**B**	**SE**	**OR**
Individualism/collectivism	−0.01^**^	0.00	0.99	0.01	0.00	1.01	0.01	0.00	1.01	0.01	0.00	1.01
Indulgence/restraint	−0.01	0.00	0.99	−0.00	0.00	1.00	−0.01	0.01	0.99	−0.03^**^	0.01	0.97
Traditional/secular values	0.43^**^	0.09	1.54	−0.28^**^	0.07	0.75	−0.67^**^	0.16	0.50	−0.29	0.13	0.75
Survival/expression values	0.08	0.08	1.08	0.10	0.07	1.11	0.13	0.13	1.14	0.50^**^	0.14	1.65
Intercept	−0.63^*^	0.22	0.53	0.47^*^	0.17	1.60	−3.15^**^	0.31	0.04	−2.00^**^	0.30	0.14
Nagelkerke R2 (%)	3.61			0.92			2.39			3.7		

* *p < 0.05*;

** *p < 0.01 (Bonferroni correction); OR, Odds ratio*.

a *Cultural dimensions and values are bipolar. Positive B values indicate higher individualism and indulgence, and higher secular and expression values; Some countries were excluded from analyses because less than five participants cited the subcategory*.

1 *Mexico and Argentina*.

2 *Norway*.

It is nevertheless, important to notice that the proportion of variance explained by cultural dimensions for each of the happiness definition categories was substantially low, ranging from 0.92% (psychological definitions) to 5.59% (family). More specifically, these dimensions explained about half of the percentage of variance accounted for by country membership.

#### Patterns and strength of relations between countries and happiness definition categories

Patterns and strength of relations between happiness definitions categories and country membership were explored through standard correspondence analysis with symmetrical normalization. The variable “Domain Definitions” comprised the definition categories described in the previous sections, while the variable “Country” included the nations (and two states for India) in which data were collected. As per the standard procedure, the unit of analysis was the number of answers classified in each category. In a preliminary phase aimed at detecting outliers, leisure showed both high absolute co-ordinate values and high contributions. Therefore, in the following steps it was treated as supplementary category, not contributing to determining the nature of the principal axes.

**Figure 3:**
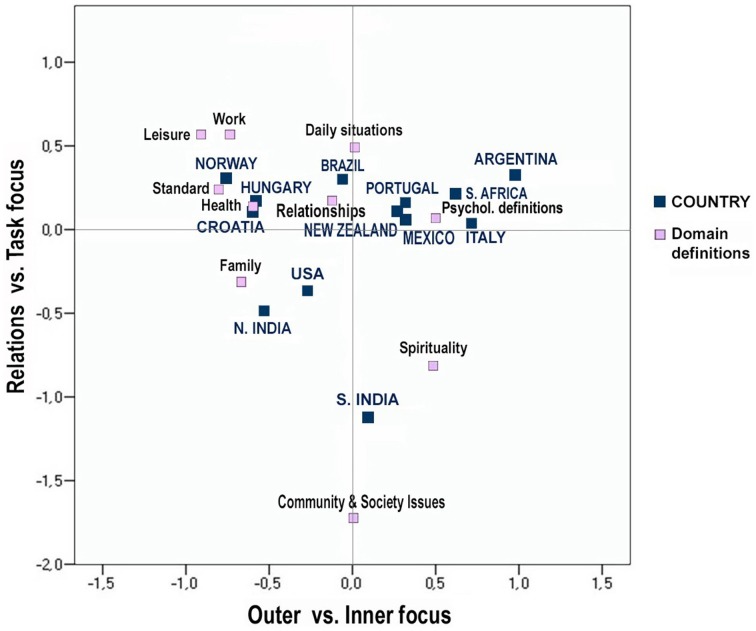
**Correspondence map based on domain-related definitions of happiness and country**.

The relationship between happiness definitions and country affiliation was significant (χ^2^ = 980.31, *p* < 0.01; Cramer's *V* = 0.13). Three dimensions could be retained based on the scree test, whose singular values were 0.27, 0.16, and 0.11 respectively. Two of these dimensions accounted for 74% of inertia (55 and 19%), and they were thus used to build a two-dimensional space containing happiness domains and countries, presented in Figure 3. Psychological definitions provided the strongest contribution to the inertia of the first dimension (41%), followed by family on the opposite side (27%). This dimension was labeled as *Outer vs. Inner focus*, ranging from active and interactive behaviors to reflection and introspection. The second dimension was best described by community and social issues (55% of inertia), and to a lesser extent spirituality (12%), and family (10%). Work followed in the opposite quadrant (10% of inertia). The second dimension was thus, named as *Relation vs. Task focus*, covering the continuum from other-oriented to task-oriented behaviors. Family showed the highest correlation between the two dimensions (−0.26). Argentina and Italy predominantly contributed to define the first dimension in the inner orientation (with 23% and 16% of inertia respectively), followed by Norway, located toward to the outer focus pole (13%). The second dimension was best explained by South India, contributing to inertia with 67% on the relational side. Norway was characterized by the highest correlation between the two dimensions (outer and task focus: 0.30).

### Relationships between socio-cultural features and psychological definition subcategories

#### Demographic predictors

Binary logistic regressions allowed us to identify demographic predictors of the subcategories of psychological definitions among participants who provided at least one answer within this domain (*N* = 1899). As illustrated in Table 7, outcome variables were represented by the most frequent subcategories: harmony, satisfaction, positive emotions, meaning, awareness, positive states, and absence of negative feelings. Countries with less than five participants providing answers in a specific subcategory were not included in the regression. The remaining subcategories were not analyzed, due to the marginal number of participants mentioning them across samples. Predictors were demographic variables (age, gender, marital status, having religion) and country membership. Compared to baseline models, all logistic regressions significantly improved prediction. The models explained from 4.40% (meaning) to 6.78% (harmony) of the outcome variance.

Table 7**Binary logistic regression: demographic and country predictors of psychological definition subcategories**.

**Table:** 

**Predictors**	**Harmony**			**Satisfaction**			**Positive emotions**			**Meaning^b^**		
	**B**	**SE**	**OR**	**B**	**SE**	**OR**	**B**	**SE**	**OR**	**B**	**SE**	**OR**
Age (30 years)^a^	0.01	0.01	1.01	−0.02	0.01	0.98	0.01	0.01	1.01	−0.03^*^	0.01	0.97
**GENDER (FEMALE)^a^**												
Male	−0.29^**^	0.10	0.75	−0.04	0.11	1.04	−0.12	0.12	0.89	−0.04	0.18	0.96
**EDUCATION (SECONDARY)^a^**												
Tertiary	0.09	0.10	1.09	0.27	0.11	1.31	0.09	0.12	0.91	0.01	0.18	1.01
**MARITAL STATUS (SINGLE)^a^**												
Married	−0.04	0.14	1.04	−0.13	0.15	0.88	−0.20	0.16	0.82	0.45	0.28	1.56
Cohabiting	0.10	0.20	1.10	−0.13	0.23	0.88	−0.23	0.24	0.79	0.14	0.38	1.15
Divorced/ widowed	−0.29	0.19	0.75	−0.04	0.22	0.96	−0.08	0.22	0.92	0.39	0.38	1.47
Religion (none)1												
Having religion	−0.01	0.14	0.99	−0.02	0.16	1.02	0.13	0.17	1.13	−0.10	0.25	0.90
**COUNTRY (PORTUGAL)^a^**												
Italy	0.46	0.21	1.58	−0.43	0.23	0.65	−0.96^**^	0.27	0.38	−0.32	0.40	0.72
Norway	−0.53	0.25	0.59	−1.88^**^	0.37	0.15	−0.11	0.28	0.90	0.16	0.41	1.18
Croatia	−0.93^**^	0.27	0.40	0.44	0.25	1.55	−0.8^*^	0.32	0.44	0.05	0.42	1.05
Hungary	0.51	0.25	1.67	−0.73^**^	0.29	0.48	−0.53	0.31	0.59	0.54	0.40	1.72
USA	−0.08	0.24	0.92	−0.42	0.26	0.66	−0.43	0.29	0.65	−0.14	0.44	0.87
Mexico	−0.76^**^	0.25	0.47	−0.70^*^	0.26	0.50	−0.59	0.28	0.56	−	−	−
Argentina	−0.09	0.22	0.92	−1.13^**^	0.27	0.32	−0.54	0.26	0.58	−	−	−
Brazil	−0.56	0.23	0.57	−0.51	0.24	0.60	−0.30	0.26	0.74	−1.07	0.52	0.34
South Africa	−0.37	0.22	0.69	−0.45	0.24	0.64	0.18	0.24	1.20	0.45	0.35	1.57
North India	−0.23	0.25	0.80	−0.99^**^	0.30	0.37	−1.13^**^	0.36	0.32	−0.73	0.53	0.48
South India	0.47	0.23	1.61	−0.63	0.26	0.53	−0.77	0.29	0.46	−0.75	0.49	0.47
New Zealand	−0.04	0.22	0.96	−0.42	0.24	0.66	0.25	0.24	1.29	0.18	0.37	1.19
Intercept	−0.10	0.32	0.90	−0.74	0.36	0.48	−0.74	0.38	0.48	−2.19^**^	0.59	0.11
Nagelkerke R2 (%)	6.78			6.69			4.83			4.40

**Table:** 

**Predictors**	**Awareness**			**Positive states^b^**			**No negative feelings^b^**		
	**B**	**SE**	**OR**	**B**	**SE**	**OR**	**B**	**SE**	**OR**
Age (30 years)^a^	0.00	0.01	1.00	0.01	0.01	1.01	0.01	0.01	1.01
**GENDER (FEMALE)^a^**									
Male	−0.18	0.18	0.83	−0.16	0.15	0.85	0.04	0.18	1.04
**EDUCATION (SECONDARY)^a^**									
Tertiary	0.38	0.18	1.47	−0.07	0.15	0.93	−0.31	0.18	0.74
**MARITAL STATUS (SINGLE)^a^**									
Married	−0.13	0.24	0.88	−0.12	0.21	0.89	−0.39	0.26	0.67
Cohabiting	−0.28	0.37	0.76	0.06	0.31	1.06	0.27	0.33	1.31
Divorced/ widowed	0.13	0.32	1.14	−0.06	0.29	0.94	−0.07	0.35	0.93
Religion (none)1									
Having religion	−0.04	0.26	0.96	0.17	0.23	1.19	0.02	0.24	1.02
**COUNTRY (PORTUGAL)^a^**									
Italy	0.62	0.39	1.87	−1.19^**^	0.33	0.31	−0.13	0.43	0.88
Norway	0.08	0.50	1.08	−0.77	0.35	0.46	0.72	0.41	2.05
Croatia	0.40	0.47	1.49	−	−	−	0.45	0.45	1.56
Hungary	0.00	0.52	1.00	−0.33	0.35	0.72	−0.45	0.55	0.64
USA	−0.04	0.51	.96	−1.04^*^	0.38	0.35	0.92	0.40	2.54
Mexico	0.13	0.47	1.14	−1.24^**^	0.38	0.29	−	−	−
Argentina	1.19^**^	0.38	3.27	−	−	−	−	−	−
Brazil	−0.46	0.52	0.63	0.37	0.26	1.45	−0.74	0.55	0.48
South Africa	0.54	0.41	1.71	−0.33	0.28	0.72	0.37	0.41	1.44
North India	−0.21	0.56	0.81	−0.43	0.33	0.65	0.33	0.48	1.39
South India	−0.23	0.52	0.80	−1.74^**^	0.46	0.18	−0.14	0.49	0.87
New Zealand	−0.13	0.47	0.88	−0.69	0.32	0.50	0.85^*^	0.39	2.33
Intercept	−2.97^**^	0.61	0.05	−1.17^**^	0.47	0.31	−2.12^**^	0.60	0.12
Nagelkerke R2 (%)	4.76			7.00			5.84		

*N participants = 1899*;

* *p < 0.05*;

** *p < 0.01 (Bonferroni correction); OR, Odds ratio*.

a *Reference category*.

b *Some countries were excluded from analysis because less than five participants cited the subcategory*.

As concerns demographic predictors of specific answer subcategories, only age and gender emerged, with younger participants more frequently referring to meaning, and women to harmony. As for country membership, taking Portugal again as the reference country, differences across samples predominantly emerged for satisfaction and positive states, and to a lesser extent for harmony and positive emotions. The probability to define happiness as satisfaction was significantly lower in Norway, Hungary, Mexico, Argentina, and North India. Italians, South Indians, Mexicans and US participants were less prone to mention positive states than people living in Portugal and in most of the other countries under examination. Croatians and Mexicans were significantly less likely to mention harmony than the other participants, while Italians, North Indians and Croatians were less prone to refer to positive emotions. Compared to Portugal, living in Argentina significantly predicted the mention of awareness, and living in New Zealand the reference to absence of negative feelings. No country differences were instead detected for happiness definitions related to meaning.

#### Cultural predictors

Binary logistic regressions further allowed to investigate Hofstede's and Inglehart-Welzel's cultural dimensions and values as predictors of each subcategory of psychological definitions of happiness. As shown in Table 8, only very few significant findings were obtained, prominently referring to Inglehart-Welzel's dimension of traditional vs. secular values. More specifically, participants living in nations endorsing secular values were more prone to describe happiness as an inner state of harmony, meaning, and absence of negative feelings. In addition, participants from more individualistic countries were more likely to refer to harmony. However, the percentage of variance explained by these dimensions for each subcategory is remarkably low, ranging from 0.45% for awareness to 3.89% for absence of negative feelings. Overall, this percentage is around one-fourth of the variance explained by country membership for the psychological definition subcategories.

Table 8**Binary logistic regression: cultural dimensions and values as predictors of psychological definition subcategories**.

**Table:** 

**Predictors^a^**	**Harmony**			**Satisfaction**			**Positive emotions**			**Meaning^1^**		
	**B**	**SE**	**OR**	**B**	**SE**	**OR**	**B**	**SE**	**OR**	**B**	**SE**	**OR**
Individualism/collectivism	0.01^**^	0.00	1.01	−0.01	0.00	0.99	−0.00	0.00	1.00	0.01	0.01	1.01
Indulgence/restraint	−0.01	0.00	0.99	−0.01	0.00	0.99	0.01	0.00	1.01	−0.01	0.01	0.99
Traditional/secular values	0.01^**^	0.00	1.01	0.00	0.00	1.00	0.01	0.00	1.01	0.01^*^	0.01	1.01
Survival/expression values	−0.12	0.07	0.89	0.02	0.08	1.02	−0.03	0.09	0.97	−0.21	0.13	0.81
Intercept	−0.74^**^	0.21	0.48	−0.39	0.22	0.68	−1.45^**^	0.26	0.23	−2.58^**^	0.39	0.08
Nagelkerke R2 (%)	2.63			1.02			1.27			2.06

**Table:** 

**Predictors**	**Awareness**			**Positive states**			**No negative feelings^2^**		
	**B**	**SE**	**OR**	**B**	**SE**	**OR**	**B**	**SE**	**OR**
Individualism/collectivism	0.00	0.01	1.00	−0.01	0.00	0.99	0.01	0.01	1.01
Indulgence/restraint	0.00	0.01	1.00	−0.01	0.00	1.00	−0.01	0.01	0.99
Traditional/secular values	−0.01	0.01	0.99	−0.01	0.00	1.00	0.03^**^	0.01	1.03
Survival/expression values	−0.18	0.13	0.84	−0.09	0.11	0.92	0.29	0.14	1.34
Intercept	−2.76^**^	0.38	0.06	−1.15^**^	0.29	0.32	−2.76^**^	0.40	0.06
Nagelkerke R2 (%)	0.45			1.46			3.89		

* *p < 0.05*;

** *p < 0.01 (Bonferroni correction); OR, Odds ratio*.

a *Cultural dimensions and values are bipolar. Positive B values indicate higher individualism and indulgence, and higher secular and expression values; Some countries were excluded from analyses because less than five participants cited the subcategory*.

1 *Mexico and Argentina*.

2 *Croatia and Argentina*.

#### Patterns and strength of relations between countries and psychological definition subcategories

**Figure 4:**
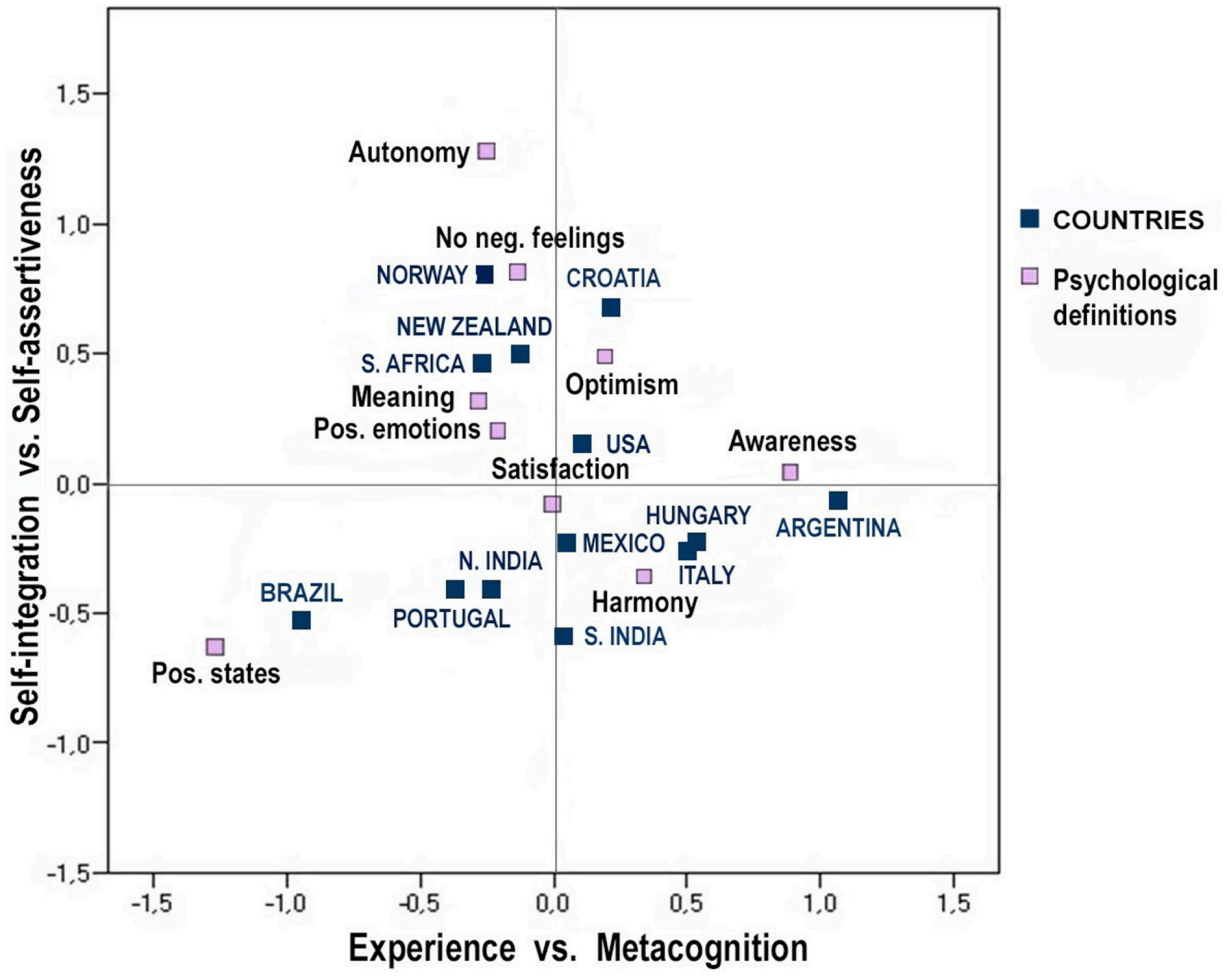
**Correspondence map based on psychological definitions of happiness and country**.

Correspondence analysis was performed on country membership and the subcategories of psychological definitions of happiness. Nine subcategories were retained, while those selected by less than 3% of participants were excluded. The relationship between psychological definitions of happiness and country affiliation was significant (χ^2^ = 463.51, *p* < 0.01; Cramer's *V* = 0.14). Two dimensions accounted for 57.2% of the overall inertia, and they were thus used to build a bi-dimensional space, as illustrated in Figure 4. The first dimension was best described by positive states (57% of the dimension's inertia) and—on the opposite side—by awareness (18%) and harmony (17%). The first dimension was thus, labeled *Experience* vs. *Metacognition*. Autonomy and absence of negative feelings showed the strongest contribution to the inertia of the second dimension (34 and 18% respectively), together with harmony (20%), positioned in the opposite side. The second dimension was thus, labeled *Self-integration* vs. *Self-assertiveness*. Satisfaction was placed close to the origin (corresponding to the average profile). Positive states showed the highest correlation between the two dimensions (−0.98).

Argentina and Brazil primarily contributed to the first dimension (32 and 23% of inertia), in terms of metacognition and experience respectively. Brazil and Norway best explained the second dimension (each with 17% of inertia), on the side of self-integration and self-assertiveness respectively. Brazil and New Zealand showed the highest correlation between the two dimensions, although in opposite directions (−0.91 and 0.80 respectively).

## Discussion

In the present study, adult participants from 12 countries in five continents were invited to define happiness in their own words. They reported a wide range of definitions, covering the biological, psychological and social spheres of individual life. While the large dataset contained a broad range of information, in line with the study aims the discussion will focus on three major topics. The first one concerns the contents of happiness definitions, through the evaluation of similarities and discrepancies between the definitions of happiness reported by the participants and those reported in the well-being literature. The second topic refers to the relation between happiness definitions and participants' demographic characteristics. The third topic concerns the relationship between happiness definitions and country/culture membership. Both nationality and culture will be taken into account, given that the 12 countries' scores cover the whole range of Hofstede's continuum of individualism/collectivism and widely differ along the two dimensions of Inglehart-Welzel's Map, namely traditional/secular values and survival/self-expressive values. The results will also be discussed in the light of the definitions of happiness provided by local dictionaries. Finally, based on the evidence of inner harmony and social relations as the most recurrent happiness definitions across countries, an overarching conceptualization of happiness will be proposed, grounded in the perspective of connectedness.

### Happiness from dictionary entries to lay psychological definitions

A growing number of studies rely on dictionary definitions of happiness to discuss variations in the use of the terms across countries. However, especially in countries using English as the second language, the semantics of words may to some extent reflects meanings adopted from the “global cultural supermarket” (Mathews, 2012) rather than culture-specific experiences (Wierzbicka, 2009). In the present study, in line with the local dictionary definitions of happiness, the majority of participants across countries defined happiness as a psychological state. In contrast, the percentage of participants reporting definitions referring to contextual categories was globally lower and varied in size across countries.

In line with the dictionaries of all the examined countries, positive emotions were frequently quoted. Related answers included the generic definitions of happiness as “a positive emotion,” “a transient feeling,” as well as reference to specific emotions such as joy, cheerfulness, enthusiasm, vitality, pleasure, comfort, being merry, and feeling energetic. In this subcategory high-arousal emotions were prominent, in line with the happiness conceptualizations predominant in both empirical studies and dictionaries' descriptions. Some country-related differences emerged as well. Within the subsample of participants providing psychological definitions, positive emotions were reported by percentages ranging from 32% of New Zealanders to 10% of Northern Indians. In line with findings obtained in Asian collectivistic countries (Yik and Russell, 2003; Lee et al., 2013), participants from North India were significantly less prone to use high arousal terms, but the same pattern was unexpectedly detected among participants from the individualistic Italy, and a similar trend was identified for Mexico and Argentina, typically considered as countries characterized by high emotional expressiveness (Ruby et al., 2012).

Satisfaction accounted for an overall percentage of answers similar to positive emotions, without variations related to demographic features. Among people who quoted psychological definitions of happiness, satisfaction was reported by percentages ranging from 48% of Croats to 8% of Norwegians. Therefore, the expectation (related to the third aim of the study) to identify personal satisfaction as the prominent psychological definition of happiness was not supported, and this was especially evident in individualistic countries endorsing secular values, such as Norway and Hungary. This finding is consistent with those obtained in the previous EHHI study. It points to an intriguing discrepancy between lay people's understanding of happiness across countries and the widespread scientific use of life satisfaction and happiness as synonyms.

In line with the expectations related to the first aim of the study, discrepancies from dictionary entries were detected for two specific definitions. In particular, happiness as luck or fortune accounted for 13 answers in total (0.17%), which were included in the miscellaneous category “daily life.” In contrast, the most frequent psychological definition was harmony, not included in dictionaries except for the Italian and Croatian ones (in the latter as peace of mind).

#### Happiness as inner harmony: an emerging psychological definition

Harmony, the most frequent subcategory within the psychological definitions of happiness, included the components of inner peace, inner balance, contentment, and psychophysical well-being. Together with awareness, it contributed to the dimension of metacognition (juxtaposed to experience) in the correspondence map referring to psychological definitions of happiness. Except for Croatia, harmony ranked first across countries, with participants' percentages ranging from 57% in Hungary to 26% in Mexico. The most frequent component was inner peace, reported by over half of the participants in Italy, Brazil, North and South India. This component includes peace of mind as well as feelings of serenity and tranquility, not included in the subcategory of positive emotions based on their low-arousal features and less transient nature. A similar distinction was made in the scale assessing durable and fluctuating happiness (Dambrun et al., 2012). Durable happiness included low-arousal feelings and it was characterized by a higher stability, in contrast with fluctuating happiness, which comprised high arousal emotions.

The component of balance was most frequent in the descriptions of Norwegian and Hungarian participants, and it referred to emotional stability, equipoise, as well as harmonization among the different aspects of the person and between the person and the environment. Contentment, measured through Cantril's ladder as synonymous with happiness in international surveys, and interpreted as the affective component of satisfaction by some authors (Rojas and Veenhoven, 2013), was primarily reported by participants from the English-speaking countries, while it was only marginally mentioned by European and South American participants (except for Portuguese ones). Psychophysical well-being, though globally less frequent than the other definitions, was quoted by similar percentages of participants across countries. It included general items referring to body/mind well-being and health.

As showed by regression analyses, gender was the only demographic feature predicting a difference in harmony frequency, and only two significant differences were identified across countries. In particular, harmony was less likely to be quoted by citizens of two collectivistic countries, Croatia and Mexico. It could be speculated that the prominence of an outward focus may prevent individuals from searching for congruence and integration at the inner level, at least in these two countries. In addition, the significantly low reference to harmony among Croatians could be referred to the problematic socio-economic circumstances, giving prominence to the endorsement of survival values and related need satisfaction. The same trend was identified when cultural dimensions were taken into account, with individualism and secular values emerging as significant predictors of harmony. However, the substantially low percentage of variance explained by these dimensions does not allow for drawing any final conclusion. At a more general level, these findings suggest the cross-country predominance of a conceptualization of happiness as a relatively stable and harmonious interplay between physical, emotional, experiential, and reflective aspects of the person. Such a definition is much closer to the view of happiness traditionally attributed to Asian cultures than to the Western ones; nevertheless, it was shared by participants across countries largely varying in language, history and cultural features.

This finding suggests the need for expanding the theoretical reflection on happiness, including the dimension of inner harmony and balance, and exploring it through more fine-grained analyses taking into account cultural and national features. A scientific literature addressing the concept of harmony does exist indeed. However, it prominently includes studies investigating interpersonal and social harmony in East Asian contexts (Wang et al., 2014; Sawaumi et al., 2015). At the psychological level, Indian scholars have investigated balance and detachment from passions as positive inner states, referring to their own cultural tradition (Pande and Naidu, 1992; Salagame, 2004). Nevertheless, the concept of inner harmony was present in the Western tradition, described by ancient Greek philosophers like Epicure, who discussed it as *ataraxia*, and further elaborated in the following centuries (for a review, see Delle Fave, 2014). The present findings suggest that the understanding of happiness as harmony has survived in folk conceptualizations, despite its marginal role in the scientific literature, possibly due to the progressive dominance of a pragmatic and achievement-focused worldview. Only few scientific works recently investigated inner harmony in relation to happiness, and only two scales are available to assess it: the Peace of Mind Scale (Lee et al., 2013), however designed to explore this construct as a characteristic specific to Eastern Asian cultures, and the Harmony Scale (Kjell et al., 2015), validated among both Western and Asian participants. The Harmony Scale represents a promising instrument to further elaborate this construct. The items closely reflect the components detected in the present study, such as perceived harmony and balance in life, feeling of attunement with the world, and life acceptance.

### Contextual definitions of happiness: the primacy of relationships

Although contextual definitions of happiness are not explicitly considered in dictionaries, the related categories subsumed almost 58% of the answers. Overall, they were more frequent in countries sharing the Germanic language root, such as Norway, United States, and New Zealand, compared with countries speaking neo-Latin/Romance languages, such as Mexico, Argentina, Italy, and Portugal. This result is consistent with evidence obtained in the previous EHHI study (Delle Fave et al., 2013). This difference can be ascribed to the specific connotations of the Latin term “*felicitas*,” primarily understood as an inner state, from which the Spanish, Italian and Portuguese words for happiness stem. On the opposite, the linguistic root “hap” characterizing both happiness and the verb “to happen” in English, and the reference to luck and good living conditions embedded in the Norwegian term “*lykke*” (Hellevik, 2008) may partially explain the predominance of contextual definitions in these countries. This interpretation is also corroborated by the analysis of the cultural dimensions. In the present study, most of the countries speaking neo-Latin languages endorse traditional values, a feature that emerged as a significant predictor of psychological definitions of happiness. On the opposite, except for religion/spirituality, secular values emerged as significant predictors of contextual definitions covering a wide range of domains, from health and relationships to work and material goods.

Despite this general difference, and in line with the expectations related to the first aim of the study, the most frequent categories cited across countries were family and interpersonal relationships. This finding is consistent with the international literature (Burleson, 2003; An and Cooney, 2006), and with evidence obtained in some of the countries involved in this study, such as Argentina (Casullo and Castro Solano, 2001 with adolescents), Brazil (Getúlio Vargas Foundation, 2013), Portugal (Rego and Cunha, 2009), Hungary (European Value Survey, 2010), and United States (Chiasson et al., 1996 with college students). However, in line with the expectations related to the second aim of the study, variations were detected according to demographic features. Family was reported significantly more often by women, less educated people, and participants cohabiting with a partner and children either presently or in the past (being widowed or divorced).

The investigation of the relationship between happiness definitions and participants' nationality and cultural dimensions allowed to detect some unexpected differences across countries. Participants from countries endorsing secular values, such as Norway and Hungary, reported family in a significantly higher percentage than participants living in the traditional value oriented Portugal. Participants from other traditional countries such as Mexico, South Africa, and North India were aligned with Portugal as concerns the probability of mentioning family as a definition of happiness, while Argentineans were even significantly less prone to do it. Italy, a country scoring on the secular side of the Inglehart-Welzel's continuum, did not differ from Portugal as well. We propose an interpretation of these apparently counterintuitive findings based on cultural and historical reasons. In traditional societies the presence and influence of family in individual's daily life and long-term plans is somehow taken for granted as an intrinsic and almost invisible constituent of life, entailing both support and constraints (Delle Fave et al., 2011b; Mathews, 2012). As concerns the more secularly oriented Italy, the finding can be ascribed to the peculiar family-centeredness characterizing the country's social structure across centuries at various levels, from entrepreneurship to daily living organization (Kotlar and De Massis, 2013). In contrast, the high percentage of Norwegians, but also US citizens mentioning family may be explained with the higher personal responsibility for the nuclear family that, besides secularism, the combination of individualism and self-expression entails (Hofstede et al., 2010). A different interpretation is instead required for the high percentage of Hungarians mentioning family. In Hungary, the orientation toward secular values is matched with the orientation toward survival ones (European Value Survey, 2010). In a society where traditional values have lost their significance, and trust in democratic institutions and civil society is low, family relationships represent the only secure source of comfort and “fullness of life” at the social level.

Other life domains were typical of specific samples. Norwegians reported a uniquely broad variety of contextual categories, including work, health, leisure and standard of living, in line with the expectations based on the country's endorsement of self-expressive and secular values. The relevance of work can be related to the Norwegian participatory system, assigning considerable power to employees and explicitly concerned with fulfillment of psychological needs at work (Emery and Thorsrud, 1969). As concerns standard of living, answers prominently referred to financial stability, and independence rather than material possessions. The “oil economy” has offered ample possibilities for realizing material aspects of a good life, possibly contributing to the salience of this category in happiness definitions. In contrast, the limited contribution of psychological definitions is consistent with the common representation of Norwegians as “unsophisticated, but practically minded” (Eriksen, 1993) and reluctant to disclose emotions. The moderate correlation between task-focus and outer-focus patterns detected for Norway in the correspondence analyses was also consistent with this interpretation.

Reference to leisure specifically characterized Norway, US and New Zealand, in line with the expectations related to their orientation toward self-expressive values. Standard of living and health emerged in Hungary and Croatia, but for different reasons. The importance of these domains for Hungarians can be related to their survival and rational orientation, as well as their high scores on Hofstede's dimension of restraint. This interpretation is further corroborated by the extreme position of Hungary on the outer- and task focus dimensions of the correspondence analysis map. As concerns Croatia, values have changed significantly in the last decades, generating uncertainty and social instability. People are more exposed to health problems because of stress and lower availability of health services. Under these difficult circumstances, it is not surprising that Croatian participants showed the lowest probability to mention psychological definitions of happiness. People pursue goals in a hierarchical order, aspiring to freedom and autonomy only after survival needs are met, and security and freedom mediate the shift from survival values toward emancipative ones (Welzel et al., 2003).

Overall, and in line with previous EHHI findings (Delle Fave et al., 2011a), community and society issues accounted for a low percentage of answers. However, peculiar trends were detected in some samples. Over 20% of the South Indians mentioned community and society as happiness definitions. They represented the only sample positioned in the relation quadrant of the correspondence map, and substantially contributed to the inertia of the relation vs. task focus dimension. Although these findings can be related to the relatively high collectivism and traditional value pattern characterizing India as a whole, they contrast with those obtained in North India, as well as in other collectivistic countries like Brazil, Mexico and Argentina, where a negligible percentage of participants (less than 5%) referred to community issues. At the country level, this difference can be related to the above mentioned social and cultural stability characterizing Tamil Nadu across centuries, in contrast with the turbulences experienced by people living in the Northern regions. The cross-country difference can be partially ascribed to the problematic socio-economic situation currently characterizing Brazil, Mexico and Argentina, that may lead highly educated urban workers to escape from social challenges to pursue happiness through personal and independent resources and behaviors. The adaptiveness of this strategy is nevertheless questionable, as higher levels of income and education do not automatically imply higher happiness (Schimmel, 2009). A similar interpretation can be formulated for the low percentage of participants referring to community and society in South Africa. However, in this specific case the high individualism score may also play a role (Hofstede et al., 2010).

### Happiness: a matter of connectedness?

Over and above differences related to country membership, cultural dimensions, and demographic features, the findings derived from this study highlight a substantial similarity across countries in the core definitions of happiness. At the psychological level, happiness was predominantly identified as inner harmony, a balanced and positive connectedness perceived among various facets of the self. This view is consistent with the concept of integrated self (Kuhl et al., 2015) and the balanced interactive model (Wong, 2011), recently explored in relation to well-being. At the contextual level, positive and harmonious family and social relationships were described across countries as key components of happiness, in line with a vast empirical evidence.

Relational and connectivity models of well-being are getting increasing attention among researchers in the domain of psychology (Wissing, 2014). Specifically consistent with the present findings are the convoy model (Antonucci et al., 2014); the relational and situated assemblage perspective (Atkinson, 2013); the multi-level well-being model (Ng and Fisher, 2013), the model of self-expansion through relationships (Aron and Aron, 2012); and the construct of interdependent happiness, as the perception of a harmonious link with the others (Hitokoto and Uchida, 2014). Kjell's (2011) view of sustainable well-being as a process based on inner and relational harmony integrates independent and interdependent dimensions, as well as the hedonic and eudaimonic perspectives. Interconnectivity models dovetail with a relational metatheoretical ontology assuming the inter-connectedness of all things (Slife and Richardson, 2008). A “strong relationality” orientation is not only context-sensitive but also consistent with a virtue ethics perspective, in which the fact-value split is transcended and the interconnectedness of all things assumed (Fowers, 2012; Richardson, 2012). From this perspective, the individual is considered a nexus of relations.

Across disciplines, harmonious integration and interconnectedness are considered the basic prerequisites for the optimal functioning of living systems—from unicellular organisms to human communities (Delle Fave and Soosai-Nathan, 2014). The fundamental interconnection and interdependence of any element in the universe is a basic tenet of quantum physics (Feynman et al., 1965; Jayasundar, 2013). At the neurophysiological level, inter-connectedness and complexity patterns were empirically identified as the core components of consciousness (Casali et al., 2013). From an evolutionary perspective, the need to belong often prevails on self-preservation needs, due to its survival and reproduction benefits (Baumeister and Leary, 1995; Sedikides et al., 2006). Social learning and transmission of symbolic information represent the basic mechanisms of cultural development (Jablonka and Lamb, 2014). Beyond conceptual and ritual differences, all religious and philosophical traditions identify the highest stage of human development with the transcendence from the individual self, by acknowledging its interconnection with a broader and more complex reality. The findings from the present study suggest that, across individualistic and collectivistic countries varying in their value orientation, harmony represents the core feature of happiness in its individual and social manifestations, as it presupposes connections or bonds at the intra and interpersonal levels.

### Strengths and limitations

A major strength of this study is represented by the large multinational sample of adult participants, balanced by demographic features and covering a reasonably wide age range and related life stages, from employment and family building to retirement and grand-parenthood. The demographic homogeneity of the local samples allowed for interpreting similarities in light of the condition of highly educated urban citizens shared by all the participants. At the same time, differences among samples could be related to cultural dimensions specifically characterizing each group. At the methodological level, the bottom-up approach and the collection of qualitative data gave voice to the participants as active producers of happiness definitions, contextualized in real life and *Zeitgeist*, in contrast to what happens with quantitative inquiries, based on researchers' assumptions and expectations (Denzin and Lincoln, 2000). The findings obtained through this study may contribute to the development of more culture-fair psychological models and constructs, by highlighting the need for verifying theoretical and often culture-driven assumptions in the context of real life, considering lay people as active co-construers of their own culture (Vaalsiner, 2007). In particular, the partly unexpected similarities and differences detected across countries in the definitions of happiness may contribute to a more culture-sensitive approach to the study of well-being (Hardin et al., 2014).

This study has several limitations as well. In spite of the above described advantages related to the homogeneous socio-demographic features of the sample, in some of the examined countries these features are not shared by the majority of the population, living in rural areas, facing economic hardship and limited access to education. Thus, some categories of happiness definitions that were recurrent in the present investigation may be relevant for particular socio-demographic groups, but not for other ones. A higher diversification of samples in terms of demographic features could allow for better disentangling the differences that were detected among countries in happiness definitions, confirming them (and thus their relationship with cultural dimensions) or providing a more articulated perspective by highlighting differences in happiness perception across social groups within the same country (and possibly across countries). Moreover, most of the samples shared a Western cultural origin: this was true of Europeans and North Americans, but also Latin Americans and South Africans. These findings therefore, cannot be generalized, especially as concerns the African and Latin American groups included in the study. Finally, countries and cultures represent conceptual and empirically different entities that do not overlap. The findings discussed here derive from cross-national rather than cross-cultural comparisons, though in some cases the historical and linguistic contiguity may allow for more culture-based interpretations.

### Future directions

This study was aimed at exploring lay adults' definitions of happiness across countries, focusing on their relationship with participants' demographics and country characteristics. Overall, a substantial similarity emerged across countries as concerns the prominent definitions of happiness, represented by inner harmony at the psychological level, and relationships at the contextual one. This finding may represent a starting point for broadening the focus of well-being research, by including the still overlooked psychological construct of inner harmony, and for building a shared conceptual background for the understanding of human optimal functioning beyond cultural specificities.

National and cultural differences emerged as well, though related to more specific contextual and psychological definitions. They were discussed and interpreted through the lens of the cultural dimensions of individualism/collectivism (Hofstede et al., 2010) and the value-related dimensions identified in the World Value Survey (Inglehart-Welzel Cultural Map of the World, 2010).

Considering the limitations of this study in terms of samples' demographic and cultural features, further unraveling of cultural similarities and differences in notions of happiness is necessary. The notion of ideal affect differs across and within countries, and it influences how lay people and scientists define happiness, how people respond to positive events, how they regulate their positive emotions, and how they interpret smiles (Tsai and Park, 2014). As most people now live in multi-cultural societies, a deeper understanding of cultural notions of happiness and well-being will be valuable to promote harmonious existence and well-being for all diverse groups within the same country.

Finally, the convergence of our findings with the growing attention to the concept of connectedness across sciences and research domains may pave the way to the development of an integrated and interdisciplinary view of well-being. The adoption of interconnectedness as a shared element could foster an authentic bio-psycho-social view of health, claimed by the World Health Organization since its foundation but hardly actualized in research and practice.

## Author contributions

ADF, IB, and MW provided substantial contributions to the conception and design of the work; the acquisition, analysis, and interpretation of data; drafting the work and revising it critically for important intellectual content; final approval of the version to be published; and agreement to be accountable for all aspects of the work in ensuring that questions related to the accuracy or integrity of any part of the work are appropriately investigated and resolved. UA, AC, TF, MH, PJ, TM, HN, JN, KS, and LS, provided substantial contributions to the acquisition and interpretation of data; revising the work critically for important intellectual content; final approval of the version to be published; and agreement to be accountable for all aspects of the work in ensuring that questions related to the accuracy or integrity of any part of the work are appropriately investigated and resolved.

## Funding

Financial support was provided to some of the authors by the University of Rijeka, Croatia (project 13.04.1.3.05); the Hungarian Scientific Research Fund (OTKA; grant PD 105685) the Victoria University of Wellington, New Zealand; the Department of Psychology, University of Oslo, Norway; the National Research Foundation of South Africa (CPRR 13092547210-91557 and RN60571-NRF IPRR-UID 85649); the Department of Psychology, Claremont Graduate University, USA; the Portuguese Foundation for Science and Technology and the Portuguese Ministry of Education and Science through national funds and co-financed by FEDER under the PT2020; UNAM-PAPIIT IG300415 Mexico (UID/PSI/01662/2013).

### Conflict of interest statement

The authors declare that the research was conducted in the absence of any commercial or financial relationships that could be construed as a potential conflict of interest.
