# Supplementary material for: Lay Definitions of Happiness across Nations: The Primacy of Inner Harmony and Relational Connectedness
Source: Front Psychol. 2016 Jan 26;7:30. doi: 10.3389/fpsyg.2016.00030 (PMC4726797; doi:10.3389/fpsyg.2016.00030)
Supplement: Supplementary file 1 [file DataSheet1.docx]

Appendix A. Dictionary definitions of happiness across countries

| **Country** | **Source** | **Original Term** | **Dictionary entry** |
| --- | --- | --- | --- |
| Portugal | Ollar, M. S. (Ed.) (2011). Dicionário do Português Atual Houaiss, s/l . Lisbon: Círculo de Leitores e Sociedade Houaiss-Edições Culturais Lda. | Felicidade | 1. Quality or state of happy; fully satisfied state of consciousness; satisfaction, contentment, well-being. 2. Good fortune; luck. 3. Positive outcome, achievement, success |
| Italy | Vocabolario Treccani (www.treccani.it/vocabolario) Istituto dell'Enciclopedia Italiana. | Felicità | 1. State and feeling of a person who is happy: a full, whole happiness; a serene, pure, tranquil happiness, happiness without shadows; to aspire to happiness; to find happiness on earth; to enjoy, savour moments of happiness; eternal happiness, heavenly bliss; Happiness! to a person who sneezes (obsolete). With a meaning closer to “joy”: he felt an intimate happiness; ironically, referring to something annoying, troubles etc. 2. Opportunity, convenience, in general the quality of an excellent outcome: happiness of a sentence, expression, idea. |
| Norway | Wangensteen, B. (Ed.) (2005). *Bokmålsordboka: Definisjons- og rettskrivningsordbok*(3rd rev. ed.). Oslo: Kunnskapsforlaget. | Lykke | 1. Destiny, coincident. 2. Fortunate destiny, luck; for congratulations. 3. Good living conditions. 4. Deep and lasting sense of joy and well-being. |
| Croatia | Anić, V. (2003). *Veliki riječnik hrvatskoga jezika*. Zagreb: Novi liber.  Anić, V., Brozović Rončević, D., Goldstein, I., Goldstein, S., Jojić, Lj., Matasović, R.,Pranjković, I. (2002). *Hrvatski enciklopedijski rječnik*. Zagreb: Novi liber. | Sreća | Psychological state of complete satisfaction and peace based on inner peace of mind and harmony in relations to the outside world. 2. Totality of favorable circumstances or circumstance. 3. Something that happens to someone by chance, a chance occurrence. |
| Hungary | Pusztai, F. (Ed.) (2003). *Magyar értelmező kéziszótár.* Budapest: Akadémiai Kiadó. | Boldogság | 1. Emotional state: someone is fully transfixed by the satisfaction with his/her fate, circumstances [i.e., happy] 2. The potential of something to make someone happy [e.g., the happiness of creation]. |
| United States | *Merriam-Webster Dictionary (htwww.merriam-webster.com/dictionary)* | Happiness | 1. Obsolete: good fortune: prosperity. 2. a) A state of well-being and contentment: joy; b) A pleasurable or satisfying experience. 3. Felicity, aptness. |
| Mexico | DEM *Diccionario del español de Mexico*. Colegio de México.  (*http://dem.colmex.mx/*) | Felicidad | Mood characterized by joy and satisfaction; condition or situation that produces it: the happiness of children, "Without health there is no happiness" pursuit of happiness. |
| Argentina | Diccionario Clarín  (*www.clarín.com*) | Felicidad | 1. Inner state of a person who feels fully satisfied with her life, with a particular situation, or because she can enjoy anything she desires: What is necessary to attain happiness in our lives? / Being with you gives me happiness. / We feel great happiness in welcoming you in our home. 2. Things or events that produce happiness: To swim in this pool is happiness./ I wish you a life full of happiness.Happiness! (plural) is used to congratulate or express good wishes to somebody in a specific situation or because of something: Are they getting married? Happiness!/ Wish you a good start of the year! Happiness! |
| Brazil | Houaiss, A. and Villar, M. S.(2009). *Dicionário Houaiss da língua portuguesa*. Rio de Janeiro: Objetiva. | Felicidade | 1. Quality or state of happy; fully satisfied state of consciousness; satisfaction, contentment, welfare. 2. Good fortune; luck. 3. Good success, achievement, success. |
| South Africa | *Oxford Advanced Learner's Dictionary* (2015). Oxford University Press: Oxford.  *Pharos English Dictionary for South Africa* (2014). Pharos Online. (*www.pharosonline.co.za/)* | Happiness | 1. The state of feeling or showing pleasure (e.g., to find true happiness; her eyes shone with happiness; their grandchildren are a constant source of happiness). 2. The state of being satisfied that something is good or right.  Having or showing a feeling of pleasure or contentment (a happy smile, I feel happy today); willing (I’d be happy to help you); lucky (by a happy chance I have the key with me). |
| India - North | Shri Nawal ji (ed.) (1986). Nalanda Vishal shabad-sagar New Delhi: Aadish Book Depot. | Sukha, Khushi (currently used as synonyms) | Happiness (sukha): (i) favorable and desired experience which one wants to have always (opposite of sorrow),freedom from disease, health.  Happiness (khushi): happiness, joy, delight, feel-good. |
| India – South | Kriyavin Tarkalat Tamil Akarati (Kriyavin Dictionary of Contemporary Tamil). ISBN 81-85602-57-3. Chennai: Cre-A. | Machilchi | Machilchi: being in a state of pleasure; being fortunate; a sense of satisfaction; an experience of pleasure that emerges out of desirable outcomes. |
| New Zealand | Orsman, H. W. (Ed.) (2001). *The Reed dictionary of New Zealand English*. Auckland, NZ: Reed Books.  Deverson, T., & Kennedy, G. (Eds.) (2005). *The New Zealand Oxford dictionary*. Melbourne, Australia: Oxford University Press. | Happiness | 1. a) Fortunate; characterized by happiness; b) (of words, behaviour, etc.) pleasing 2. colloq. Slightly drunk. 3. colloq. Inclined to use excessively or at random (trigger-happy). |
